# Seismic Protection of RC Buildings by Polymeric Infill Wall-Frame Interface

**DOI:** 10.3390/polym13101577

**Published:** 2021-05-14

**Authors:** Ahmet Tugrul Akyildiz, Alicja Kowalska-Koczwara, Łukasz Hojdys

**Affiliations:** Faculty of Civil Engineering, Cracow University of Technology, 31-155 Cracow, Poland; akowalska@pk.edu.pl (A.K.-K.); lhojdys@pk.edu.pl (Ł.H.)

**Keywords:** polyurethane, flexible joint, RC building, brick infill, concrete damaged plasticity (CDP), earthquake

## Abstract

This paper is aimed at investigating the usage of flexible joints in masonry infilled walls surrounded by reinforced concrete (RC) frames. For this purpose, a real-size specimen was numerically created and exposed to the seismic loads. In order to evaluate both in-plane and out-of-plane performances of the infill walls, the system was chosen as a box shaped three-dimensional structure. In total, three different one-story constructions, which have single bays in two perpendicular directions, were modeled. The first type is the bare-frame without the infill walls, which was determined as a reference system. The second and third types of buildings are conventional mortar joint and PolyUrethane Flexible Joint (PUFJ) implemented ones, respectively. The influence of these joints on the material level are investigated in detail. Furthermore, general building dynamic characteristics were extracted by means of acceleration and displacement results as well as frequency domain mode shapes. Analyses revealed that PUFJ implementation on such buildings has promising outcomes and helps to sustain structural stability against the detrimental effects of earthquakes.

## 1. Introduction

Masonry today maintains its popularity as a construction technique and its derivative types of methods, e.g., infill wall systems are still being used widely across the globe. The main advantages of this practice could be stated as being a well-known building method, easy implementation procedure thanks to it allowing for the arrangement of blocks arbitrarily and the possibility of utilizing the large spectrum of building materials, including, but not limited to, clay, earth, stone, concrete, etc. Other than that, having a variety of usage purposes, such as the implementation of either a primary or auxiliary load carrying member, insulation, as well as architectural design options also makes the masonries an attractive alternative among the other construction methods.

**Figure 1:**
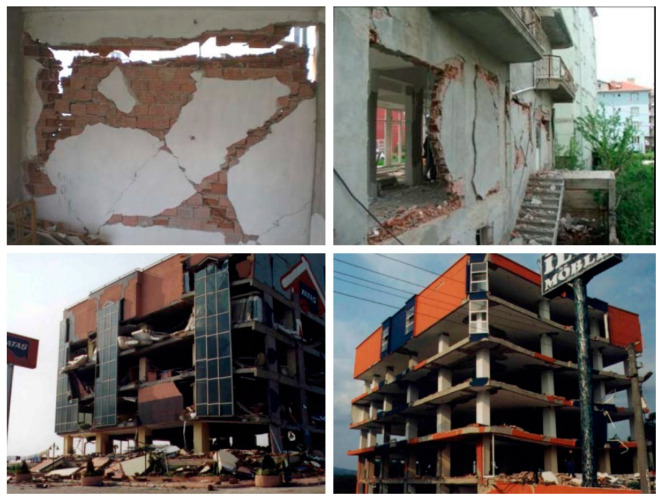
Infill wall damages from Simav, 2011, (**top**) [5] and Kocaeli, 1999, (**bottom**) [4] earthquakes.

While having many advantages, masonries have major drawbacks in some certain cases. These walls consist of multiple different units, such as bricks and mortar, which are thus prone to exhibit complex behavior under particular dynamic loads, e.g., earthquakes. The elements are very often created by the materials that have limited deformation capacities and cannot therefore satisfy the high displacement requirements for such forces. In particular, when the masonries are utilized as infill walls in the reinforced concrete (RC) buildings, interaction effects emerge between the frames and walls, which potentially cause damage to both these members due to the lack of ductility around the boundary zones, as well as the intrinsic material characteristics [1,2,3]. As a result of cyclic continuous loads, slight damages become visible and expand through the walls and RC frames, which eventually lead to either local or total structural instabilities. Past earthquakes show us the infill wall related damages in Figure 1 [4,5]. Various crack and fracture scenarios are seen in the real-life examples of the damaged masonry walls. However, it is still possible to categorize these in two main groups: in-plane and out-of-plane damages. For the in-plane failures, dominant forces occur inside of the wall plane and damages take place when the stress carrying capacity of the wall members is exceeded. Diagonal cracks or corner crushes are typical examples of this type of failure. In the case of the wall units comprised of relatively stiff materials, such as strong bricks, it is known that RC frames could also become damaged. Besides, out-of-plane failures indicate disturbance in the direction perpendicular to the wall plane. It is mainly observed when the seismic excitations cause damages to the weak connections between RC frames and infill walls. It is also possible that both types of failures could be effective progressively or simultaneously on the same wall. This is a matter of loading and construction features.

However, infill walls can protect buildings from the collapse scenarios and can also save the lives of people, or at least give them time to evacuate the building right before the disaster [6]. In the article by Albayrak et al. [6], authors invoke three limit conditions of damage: minimum damage limit, safety limit and collapsing limit. It is very important to extend the time in the first two limits. In Reference [7], the authors also mentioned the possible positive effect of non-structural elements on the behavior of structures during an earthquake. Some of the authors [8,9] indicated the impact of irregularities on the structure of its seismic characteristics.

During an earthquake, a large amount of energy is firstly received by the foundation and after that it is distributed to the other structural members. Construction systems can often be highly complex due to the configuration of members and their heterogenous material and unit features. Therefore, it becomes a rigorous work to predict the exact failure zones in a building. While the developed technology and our engineering knowledge help us to design and create preventative solutions against earthquakes, and infill wall effects other than their mass loads are currently either omitted completely [10,11,12] or taken into the consideration partially by means of diagonal implicit strut models [13,14]. Design codes have also started to cover this subject recently [15,16,17]. In light of the aforementioned information, it can be said that masonry damages in RC buildings are mainly the result of interaction effects occurring between the frames and infill walls. However, current modeling strategies cannot comprehend the actual behavior of infill wall damages, which can drastically change the building dynamic characteristics, not only in a local zone, but entirely [18,19,20]. Several researches have touched on this subject previously. Among these, Preti et al. [21] suggested using multiple horizontal or vertical sliding joints located on the different coordinates through either the wall height or length. Totoev and Al Harthy [22] tested a dry stack mortarless system called semi-interlocking masonry, which aims to dissipate seismic energy as a passive system. It requires specially produced block units for constituting the walls. Misir et al. [23] conducted a similar study and tested the in-plane drift performance of concrete frames with the notch provided hollow clay blocks named as locked bricks. Vertical gaps were provided on the side of walls, thus larger drift capacities were intended without compromising the infill stability. In another study, Vailati and Monti [24] replaced the traditional mortar with plastic joints aiming to reduce the earthquake-induced interaction effects between the masonry block units as well as with the surrounding frames. Thus, the plastic bed joints were claimed to provide more ductile systems that can resist safely against the seismic loads in return for less contribution to the overall stiffness of buildings. Johnston et al. [25] performed shake table laboratory experiments on half-scale pre-cast concrete frames comprised of timber infill walls. Aluminum channels and gypsum boards were used as connectors in order to provide the little interaction forces with the surrounding frame. Since the primary aim of their study was to test a building against the design level earthquakes, excessive drifts were omitted, and therefore the top drift ratio limit was set as 1.6%. Tasligedik et al. [26] studied a low damage solution technique on drywall partitions by means of implementing modifications to the standard connection detailing. In their research, the infill walls were totally isolated from the rest of the structure, since gaps were provided on the boundaries and therefore the aim was to enhance the drift capacity rather than increasing the total stiffness of the system. Other than that, polymer-based solutions have started to find an area of usage in civil engineering problems. Fiber reinforced polymer (FRP) is one of the popular ones that can be used as wrapping sheets in order to enhance the strength and durability of a variety of structural members, particularly columns and piles. It is known that glass-FRP (GFRP) systems are already in use globally and are effective in extending the service lives of concrete, steel and timber structures [27]. Thanks to its versatility, GFRP offers alternative solutions to be used in a large spectrum, such as for designing composite railway sleepers [28] or water-retaining wall assemblages [29]. However, the carbon-FRP (CFRP) method is generally preferred in seismic resistant design solutions, since more confinement pressure is possible to be achieved in this technique due to their higher mechanical properties compared to GFRP [27]. In particular, for the infill masonry walls, CFRP-made diagonal strips are used, which have the potential to enhance the drift and strength capacities of the frames [30,31,32].

It can be seen that the majority of these studies either focus on developing joint systems in order to provide intentional wall detachment by special connector solutions or using composite strips for wrapping the walls, which can potentially increase the infill strengths. For the connector solutions, drift capacity can be increased, however possible strength contribution of the infills are waived. In the latter case when FRP strips are used, the strength phenomenon is also taken into consideration. However, the implementation of such a solution requires harder workmanship, since the strips should be properly bonded to the surrounding frame. This is not always a feasible solution, especially when the frame configuration has restrictions, such as in the case of hidden beams, where it is a challenge to find a strong frame for the bonding zones. Therefore, an innovative solution is proposed in this paper that uses a polymer-based material as a joint member between the masonries and the RC frames. They are called PolyUrethane Flexible Joints (PUFJ) and have already been tested for various purposes in the past [33,34,35]. Large deformability capacity, together with the high bonding strength, makes PUFJ an alternative solution that can protect both infill walls and RC frames against earthquakes. Some large-scale laboratory experiments are already being done for this purpose and the preliminary results regarding the effectiveness of the material can be found elsewhere [36,37]. According to the in-plane quasi-static tests conducted in Reference [36], it is shown that PUFJ implementation considerably increases both the lateral load carrying capacity (35% higher compared to traditional method) and maximum drift limits (reaching above 4.4%, whereas in the case that the stiff joint is used, this value is only about 1.6%). This outcome denotes a vital principle in terms of the seismic resistant design that ductility, as well as strength capacities might be improved by the PUFJ. Shake table experiments in Reference [37] point out another fact that usage of PUFJ enables sustainable building stability of a real-size three-dimensional specimen against the large seismic excitations (maximum base acceleration above 1.5 g). In addition, the most recent studies have revealed that the derivations of this material can be used effectively as a quick seismic protective intervention for the structural members [38] and these are durable against extreme outdoor conditions [39].

This study is focused on the numerical modeling of such an application on a three-dimensional structure. In the literature, many of the detailed numerical analyses regarding the infilled frame topic are conducted on two-dimensional space and particularly monotonic or cyclic quasi-static loading regimes are followed. Therefore, inertia effects or out-of-plane behaviors cannot be comprehended completely. However, three-dimensional models are primarily intended to create solutions for design practitioners and thus infill walls are simulated with rough and simplified methods, such as strut modeling. Since the primary concern of this study is to investigate the earthquake performance of PUFJ material, the inertia effects should be considered during the analyses. Therefore, a three-dimensional model was preferred. In addition, rough strut models were omitted whilst modeling the walls, since the efficiency of joints could not be captured in this way. Hence, solid elements were used for the numerical analyses and the details are shared in the next section. The study is divided into two phases: in Phase I, the seismic record to be used as a base loading for the systems was taken from another analysis. In this phase, a multi-story building was designed and the relevant acceleration data was extracted from it. For Phase II, multiple steps were followed: firstly, different materials that were to be used in the analyses were created numerically and tested individually. Once the relevant calibrations were made, a simplified method was later adapted for the wall design. Following that, three types of buildings with an identical RC frame and loading details were created. The first type was determined as a reference system without the infill walls and were named Bare-Frames (BF). The second and third types represent the traditional mortar and PUFJ implementations as a joint material along the frame-masonry boundary zones, respectively. The results are given and compared here on the individual element basis, as well as on the global dynamic characteristics. Details are provided in the following sections.

## 2. Phase I Details

**Figure 2:**
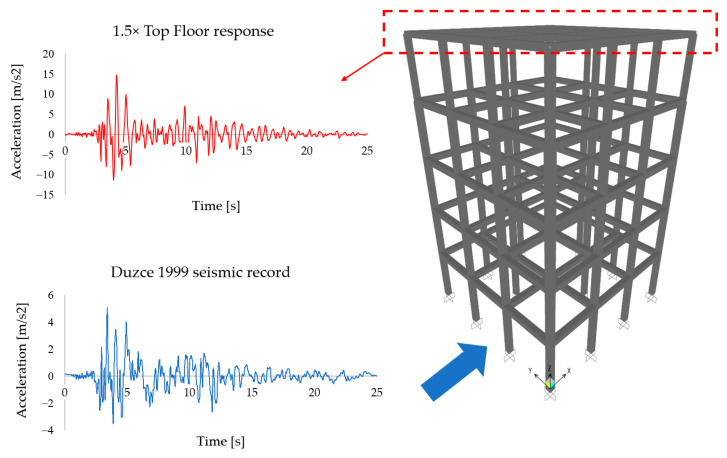
Details of the seismic records and 3D frame building view.

In order to establish a realistic seismic loading for the planned detailed analyses, a multi-storey building was first created numerically in the structural analysis program SAP2000 [40]. The hypothetical building consists of five storys and was assumed to have fixed supports on the ground. All beam and column members were modeled by one-dimensional bar elements. Nonlinear behavior of the frame building was provided by means of assigning plastic hinges at both ends of the beams and columns. The seismic acceleration data was taken from the literature (Duzce, 1999 earthquake, Figure 2). The loading was implemented on the ground level and in one direction only, since the building was modeled symmetrically. After evaluating the results, it was decided to take the acceleration response of the top-level storey for further analyses. It is due to the fact that top levels naturally have the highest acceleration outcomes and are therefore exposed to earthquake effects more severely. Moreover, preliminary Phase II analyses indicated that even the top-storey results could not exhibit the worst scenarios, hence the top-storey acceleration response was increased by multiplying the data by a factor of 1.5. This new acceleration data was used in the next phase of analyses, see Figure 2.

## 3. Phase II Details

In Phase II, the finite elements method, based on the Abaqus [41] program, was used throughout the numerical analyses. Three different buildings were modeled in this phase. The reference system had no infill walls and was named as Bare-Frame (BF). The second type had infill walls in all perimeters and bonding between the walls and RC frames was provided by traditional mortar (TM), therefore named as the TM type of building. The third and last type had identical features of a TM one, except the only difference of bonding material. PolyUrethane Flexible Joints (PUFJ) were used for this type.

**Figure 3:**
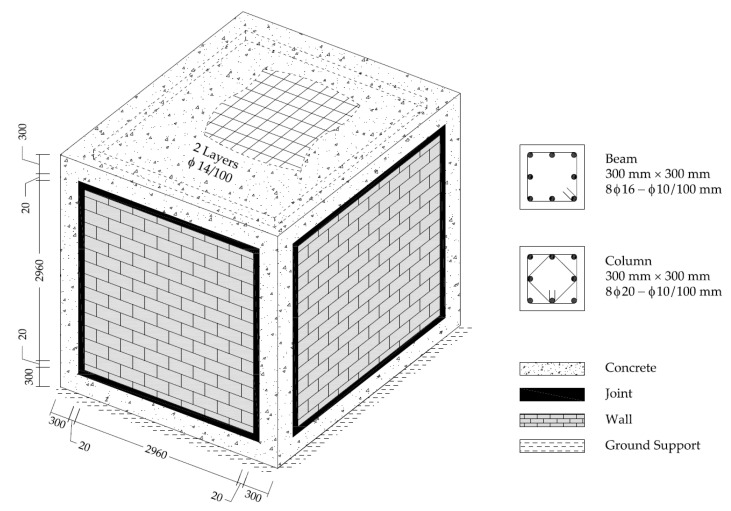
Schematic view of the large size buildings [mm].

The buildings were created to reflect the real-size dimensions and had cubic shapes. All these had fixed ground supports on the bottom-level, with the only translational motion exception of freedom in the earthquake loading direction. Out-to-out distances of the width in both directions and height were determined as 3600 mm. The RC frame consists of a square-shaped beam and column elements with the dimensions of 300 mm on each edge. A concrete slab on the top level was designed to distribute the vertical loads and was configured to have a 150 mm thickness. The clear span between each column and the top to bottom beams were equal to 3000 mm. These rectangular voids were filled by 65 mm thick masonry walls for the TM and PUFJ models. It is worth mentioning that the walls were designated to act as merely non-load bearing parts of the structure, following the common design practice for such systems. Therefore, some aspects of the design criteria set by the relevant building codes, such as slenderness ratio for the load carrying walls, is neglected. This approach enabled us to accelerate the run time for the further analyses, due to the fact that substantially less numerical elements were used. Moreover, joint thickness between the infill walls and RC frames was determined as 20 mm for these models. In addition, various reinforcement details were adapted for the RC members. A schematic view of the building is given in Figure 3.

While performing the analyses in the models, in addition to the gravity loads, 1000 kg/m^2^ of mass was exposed to the top slab throughout the entire seismic loading step. However, several small size specimens were first modeled and tested before starting the large size analyses for calibration purposes. In the next sections, these are explained.

### 3.1. Material Constitutive Models

Modeling in filled wall systems in RC structures required us to take into consideration multiple material properties and their combination of working together as a whole system. Typically, mortar, brick units, concrete, steel bars and respective interaction of these materials need to be defined. All of the aforementioned materials have their own characteristic mechanical features. Therefore, each material exhibits different behavior under loads. The interaction between those is also another concern, which is a complex phenomenon itself. Several techniques were used while creating the constitutive models and were explained with the details in this section.

**Figure 4:**
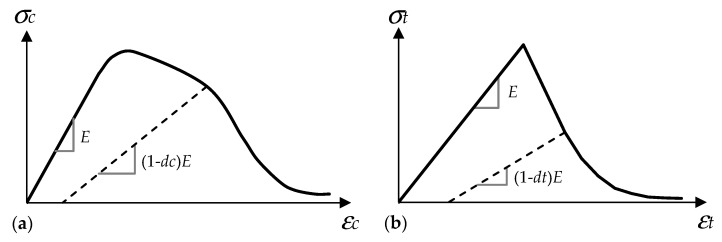
Damage evolution concept: (**a**) compression and (**b**) tension.

Concrete is considered to be a quasi-brittle material and it tends to behave elastically in a limited range of deformability. Its strength and durability capacity highly depends on the proportional mix of multiple components namely, cement, aggregate and water. Despite its heterogenous nature, in practice, it is a common approach to model the concrete with isotropic material properties, since the reliable results can still be achieved. However, compression and tension features are substantially different and thus need to be determined separately. Abaqus [41] offers multiple solutions in terms of modeling the concrete like materials. One of the popular ones is named the Concrete Damaged Plasticity (CDP) technique that is also preferred for this study. The results of some previous researches constitute the basis of this method [42,43,44]. It is suitable for monotonic, cyclic and dynamic loads. The continuum, plasticity-based damage model is followed and it enables us to model failure mechanisms for tensile cracking and compressive crushing of concrete. It is capable of providing solutions to simulate hysteretic behavior of concrete under reversal loads such as earthquakes [45], besides that different material compositions e.g., Steel or Glass Fibres are feasible to be defined in the concrete matrix with this method [46,47]. Post-damage behavior is also possible to be described using the stiffness degradation approach by means of modifying the initial elasticity modulus with a damage factor. In order to determine the damage parameters, a solution suggested by Birtel and Mark [48] was followed. In this reference, the authors declare two damage factors, *dc* and *dt,* for compressive and tensile components, respectively. Plastic strain: *εpl*, stress: *σ*, Young’s modulus: *E* and a constant value: *b* (0 < *b* < 1) are given as the required parameters, while determining the damage factors. In their research, a shear test on an RC beam was numerically simulated and the verification was made with the experimental results. The constant value of *b* for compressive and tensile loading paths were determined as *bc*: 0.7 and *bt*: 0.1, respectively. By default, this assumption was utilized in the current paper. Equation (1) shows the details of this formulation. In Figure 4, the damage evolution concept is briefly represented for compressive and tensile loading.

(1) $$d_{c,t}=1-\;\frac{\sigma_{c,t}E_{c}^{-1}}{\varepsilon_{c,t}^{pl}\left(\frac{1}{b_{c,t}}-1\right)+\sigma_{c,t}E_{c}^{-1}}$$

**Figure 5:**
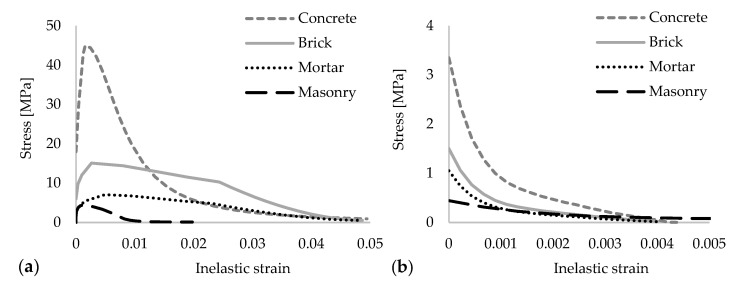
Material inelastic stress-strain curves: (**a**) compression and (**b**) tension.

For this study, the C35/45 class of concrete was used. Mechanical properties were taken as recommended in the technical codes of Eurocode-2 [49] and FIB Model Code [50] for calculating the compressive and tensile stress-strain relations, see Figure 5. Afterwards, the required CDP parameters were defined, as given in Table 1.

Since bricks and mortars also have similar quasi-brittle material characteristics as concrete, the CDP modeling technique was adopted for these as well. Experimental research outcomes of Kaushik et al. [51] were utilized as the basis of material properties definition. The material models were numerically tested and were later given in this study, while modeling the small size tests by Viskovic et al. [52]. Stress-strain curves and the input parameters are presented in Figure 5 and Table 1, respectively.

**Figure 6:**
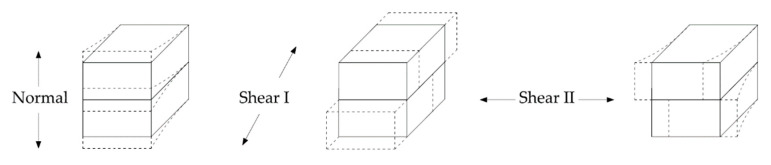
Possible damage types of the cohesive surfaces.

**Figure 7:**
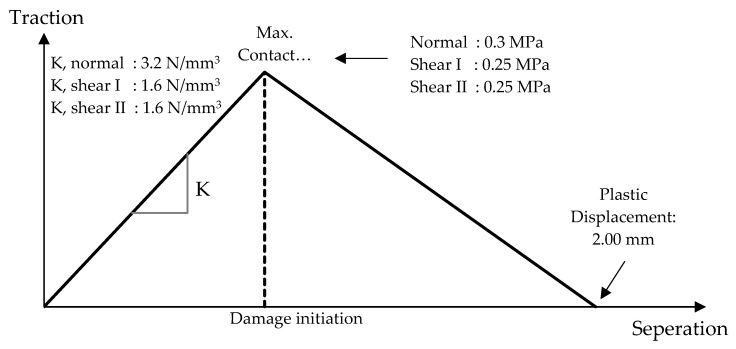
Traction-separation damage evolution.

In the micro model technique, in addition to the independent material properties, the interaction between those also need to be defined, such as brick-to-mortar or concrete-to-mortar interfaces. Of the noble solutions, that provided by Abaqus [41] was preferred in order to overcome this challenge, in which cohesive surfaces enable us to simulate compressive, tensile as well as the shear behavior of the interaction zones. This method is particularly suitable when the traction-separation kind of damage is expected on thin layer surfaces. It is capable of creating the damage models on three different directions, one to the interface normal and the other two parallel to it, as shown in Figure 6. The model requires the input of stiffness values of contact and the corresponding damage initiation-evolution parameters by means of evaluating the stress, displacement or energy values. It assumes a linear behavior until reaching the ultimate load capacity and afterwards either linear or exponential damage evolution is possible to be defined. For this study, a linear branch of damage propagation was assumed, as given in Figure 7.

Reinforcement of the concrete was provided by utilizing the various sizes of steel bars. B500B type of steel was chosen, since it is commonly used in the design practice. Its well-known elastic and plastic material properties were put as input in Abaqus and introduced to the program as embedded regions in concrete. Such an assumption is capable of demonstrating a real-like reinforced concrete behavior [53,54], however it needs to be noted that no-slip is expected in this method through the interfaces between concrete and steel bars.

**Figure 8:**
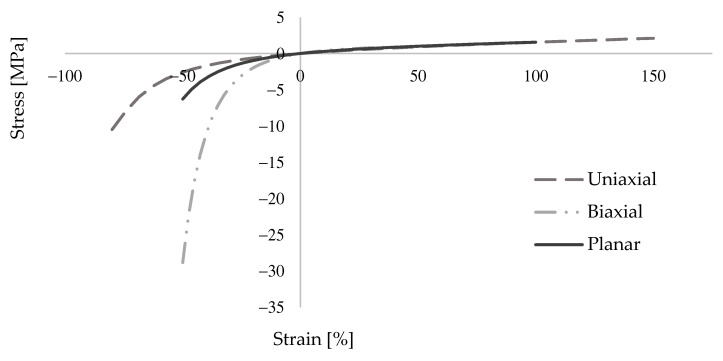
PolyUrethane PM test results from Kisiel [55].

PolyUrethane PM, which was used in the numerical models for providing flexible joints, can be considered as a hyper-elastic material. It exhibits highly nonlinear behavior and provides strain values up to 150%, yet it is still capable of sustaining its strength. Extensive research by Kisiel [55] on this topic has revealed the response of this material under different loading conditions and for several sizes of specimens. Uniaxial, biaxial and planar compression-tension tests were performed in order to find a suitable model for Abaqus, see Figure 8. Accordingly, Mooney-Rivlin hyperelasticity model was suggested to be used. Moreover, the best matching Poisson’s ratio with the test results was determined as 0.40 in that research. In this study, the data from Kisiel’s work [55] is adapted. In addition, material damping features were defined, since the dynamic behavior of large size tests was investigated, which is given with details in the next sections. Previous research was used for this purpose. Kwiecień et al. [56] found out that the damping coefficient for the polymer was 0.06. This value was also used in this study and the relevant Rayleigh parameters were calculated by following the approach in Spears and Jensen’s work [57].

Numerical models were created for representing the three-dimensional structures. Therefore, the solid (continuum) elements library provided in Abaqus [41] for the materials were used in this study. Concrete, brick and mortar were meshed with hexahedral C3D8R first-order interpolation elements, which have a single integration point and can thus provide a substantial computational gain compared to its full integration counterparts. However, second order accuracy and enhanced hourglass control options aimed to reduce the mesh distortion problems and establish more reliable results. PUFJ material was modeled in a similar manner and C3D8H hexahedral hybrid elements were preferred for this type, which is intended to model incompressible or almost incompressible materials. In contrast to the others, steel bars were created with the two-node T3D2 truss elements that can transfer the axial forces only. In terms of meshing, various mesh sizes were preferred, while modeling the different elements. This was done in a way to optimize the computational cost efficiency and the accuracy of the results. For the small size models, mesh validation for the bricks and joints was made by means of comparing the numerical results with the experiments. Therefore, multiple iterations were followed until converging with the test results. However, for the wallet and large size models, preliminary mesh sensitivity analyses were performed, which were previously adapted from the small size specimens and eventually it was determined that following values gave similar results of the relatively finer mesh sizes. For example, C3D8R mesh size was initially chosen as 40 mm and later found out that 80 mm mesh size was also able to draw similar displacement and acceleration curves on the global overall level and besides in terms of the stress diagram distribution. Therefore, 80 mm was preferred for further steps in order to accelerate the analyses. In this way, the short/long edge ratio was provided to not be less than 0.25 at any part of large size models. For the joints where the higher attention was needed, finer meshes were utilized in order to capture a more real-like behavior and therefore the mesh size of 5 mm was chosen. Finally, the one-dimensional reinforcement steel bars were meshed with the maximum size of 50 mm, hence longitudinal-stirrup bar intersections and also singular points on those free mid-spans between the intersections could be smoothly meshed.

### 3.2. Small Size Models

**Figure 9:**
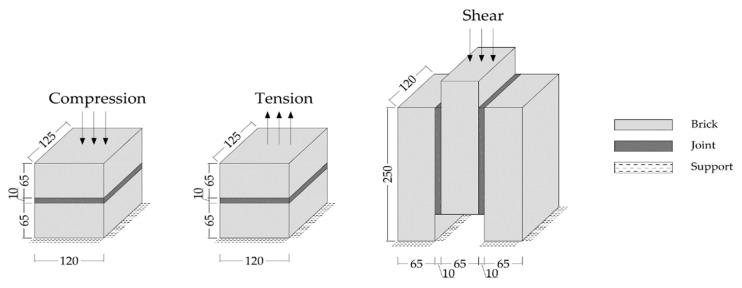
Adapted test configuration of the small size specimens [mm] from Reference [52].

**Figure 10:**
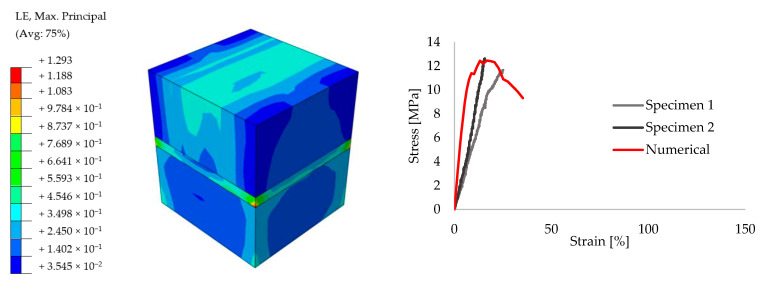
Compression results for the small size specimens with mortar: true strain diagram (**left**) and comparison of the numerical and experimental tests (**right**).

**Figure 11:**
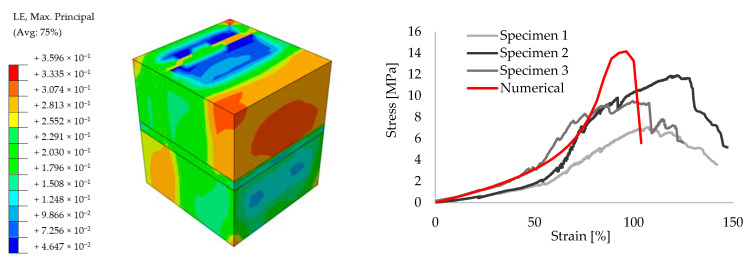
Compression results for the small size specimens with PolyUrethane PM: true strain diagram (**left**) and comparison of the numerical and experimental tests (**right**).

**Figure 12:**
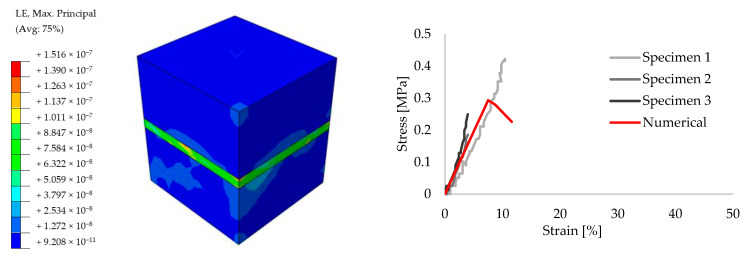
Tension results for the small size specimens with mortar: true strain diagram (**left**) and comparison of the numerical and experimental tests (**right**).

**Figure 13:**
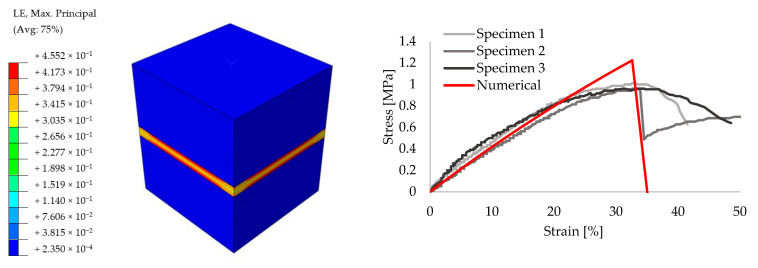
Tension results for the small size specimens with PolyUrethane PM: true strain diagram (**left**) and comparison of the numerical and experimental tests (**right**).

**Figure 14:**
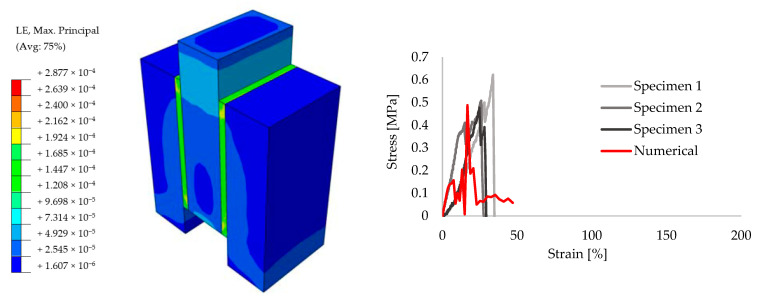
Shear results for the small size specimens with mortar: true strain diagram (**left**) and comparison of the numerical and experimental tests (**right**).

**Figure 15:**
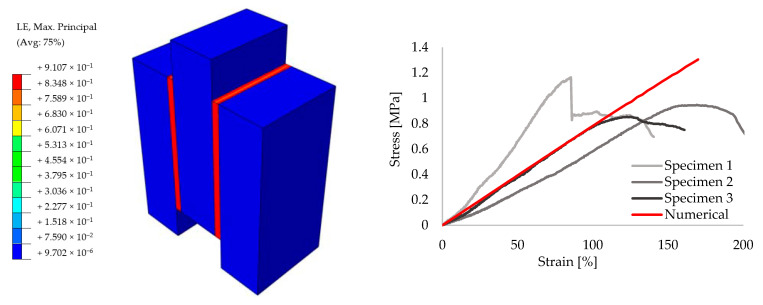
Shear results for the small size specimens with PolyUrethane PM: true strain diagram (**left**) and comparison of the numerical and experimental tests (**right**).

In order to achieve reliable results for the further analyses, preliminary small size specimens were modeled by means of creating different masonry configurations. For this purpose, experimental results from the literature are taken [52]. The small size specimens were configured by solid clay bricks with the dimensions of 65 mm × 120 mm × 250 mm and joints with 10 mm thickness. Joints were utilized by traditional mortar with the compressive strength of f_m_ = 7.2 N/mm^2^ for the stiff bonding, whereas PolyUrethane PM was chosen for providing the flexible joints. Compression, tension and shear test configurations are presented in Figure 9. The results of the experiments are compared to the numerical models. Figure 10, Figure 11, Figure 12, Figure 13, Figure 14 and Figure 15 show the final steps of analyses, together with the stress-strain curves. In the original study [52], stress values were obtained by means of dividing the applied force to the net surface area of the joints. For the compression and tension tests, this area is equal to the surface perpendicular to the loading (120 mm× 125 mm), whereas for the shear tests, the area was determined from the joints laying parallel to the shear force (2 mm × 120 mm × 200 mm), see Figure 9. However, the strain values were calculated using the joint thickness. Accordingly, shortening and elongation of the joint thickness was a matter of strains for the compressive and tensile tests, respectively. Similarly, the joint deflection was the source of strain measurement for the shear tests. It was obtained by finding the ratio between the tangential vertical displacement of joints and the length of the net bonding zone parallel to the loading.

Overall, it can be seen that reasonable convergence was achieved in the numerical models. In terms of the compression results, numerical analyses estimated the peak stress levels accurately for the mortar specimens, though the initial stiffness values were over-estimated. However, analyses with the flexible joints provided a good match during the ascending stiffness branch at the beginning, whereas the peak stress was calculated higher than the experimental measurements and rather sharper softening behavior was seen in the numerical analyses. Elastic stiffness values of the numerical results had good agreement with the experimental ones for the tensile tests of both stiff and flexible joint implemented specimens. Analysis of the mortar tests achieved a close match with the mean peak stress values of experimental outcomes, although PolyUrethane PM utilized numerical analysis slightly exhibited higher maximum stress capacity. Similarly, shear analyses were also able to simulate the real behavior in the initial elastic branch, however, the numerical analysis belongs to flexible joint tended to exhibit higher stress values at the ultimate levels.

As a next step, a previously adapted micro-model approach was compared to the macro-model technique in which bricks units, mortars and their interfaces were modeled as a single element, which reflects the isotropic mechanical features. This method is later followed, while creating the wall models for large size analyses. Such a simplification was essential, since the detailed micro models are not very feasible for the large size analyses due to the high computational requirements. Suggestions from Kaushik et al. [51] and Eurocode-6 [58] were taken into account when determining the macro model parameters, although Young’s modulus was modified differently than the aforementioned references, since the preliminary analyses gave much higher stiffness values for those. In several trials, the Young’s Modulus value of E = 1000 MPa was found to be a close match for this specific study. Wallet models were created accordingly and tested diagonally through their in-plane directions. The models were configured to utilize 65 mm wall thickness as similar to the large size specimens. Material properties and the stress-strain curves of the masonry element are given in Table 1 and Figure 5, respectively.

**Figure 16:**
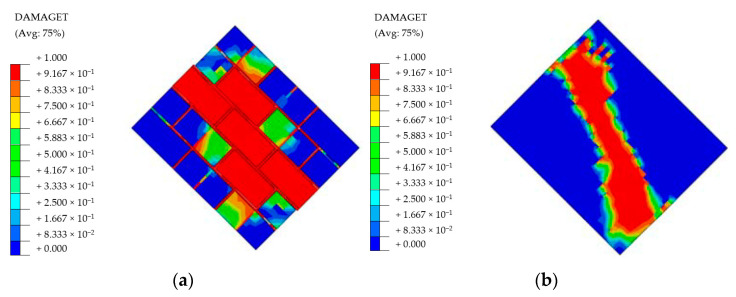
Damage distribution on the (**a**) micro-modeled wallet and (**b**) macro-modeled wallet.

**Figure 17:**
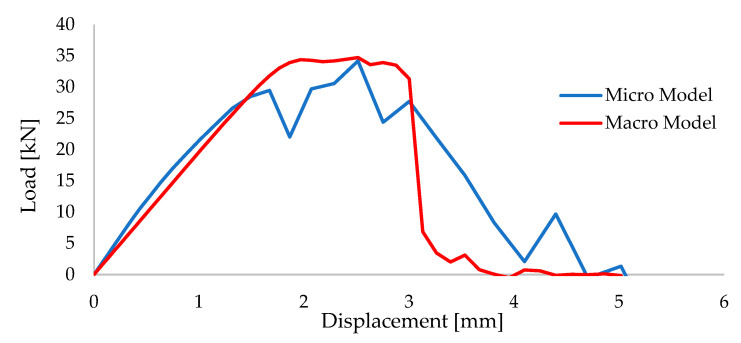
Load-Displacement curves of the micro and macro modeled wallets.

The comparison between these two modeling strategies is made in Figure 16 and Figure 17. It is an important note that such diagonal wallet tests are highly dependent on the boundary conditions. For this study, support zones were provided as 15% of the relevant edge length and no constraints were enforced on the remaining free edges. Nevertheless, this research specifically aims to investigate the bonding materials between the walls and frames. Therefore, sensitive boundary condition analysis was neglected as it is not the main concern of this paper.

### 3.3. Large Size Models

**Figure 18:**
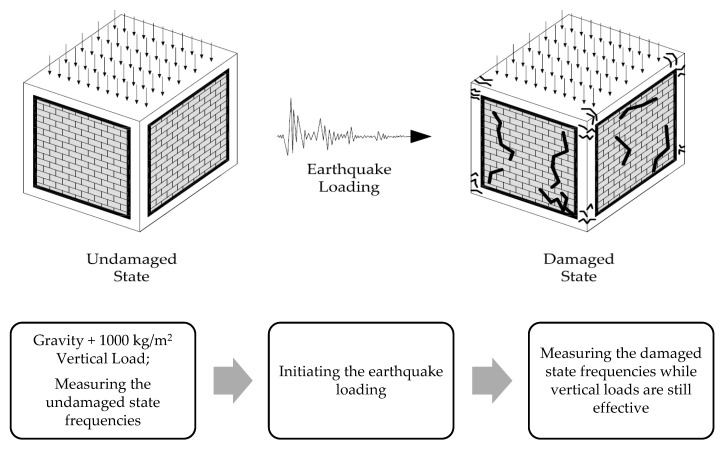
Loading and measuring procedure for the large size analyses.

In accordance with the ultimate purpose of this study, real-size cubic shaped RC in filled frames were numerically created. Data from the previously mentioned calibration tests were taken into account when modeling the large structures. All building types—BF, TM, and PUFJ—were assumed to have fixed supports to the ground at the bottom level. However, translational freedom in the earthquake loading direction was enabled in order to actualize seismic acceleration. Both material and geometrical nonlinearity (second order effects) were enabled in the analyses. The same loading procedure was followed for all the three frame types. At first, the frames were forced with the gravity loads induced by the mass of the systems and the additional vertical load on the top slab level. Once the loading step was complete, the natural frequencies of structures were measured. Following that, the earthquake loading was initiated by means of imposing the acceleration response given in Figure 2 on the ground level and in a similar approach to the last step, the modal frequency results were checked at the end of the analyses. This approach is represented by a flowchart given in Figure 18. In the next sections, the results are discussed.

### 3.4. Results

The results are evaluated in terms of the damage status of constructional elements individually, as well as the structural dynamic behavior changes holistically that occurred because of these damages. Failures due to the cracking of concrete and masonry members were taken as the primary factor of strength loss for the buildings when the element base investigation was the concern. However, the frequency shifts and the corresponding stiffness changes were assumed to be the major indicators while determining the entire structural behavior. Acceleration and displacement responses of the buildings were also reviewed.

#### 3.4.1. Material Damage Status

**Figure 19:**
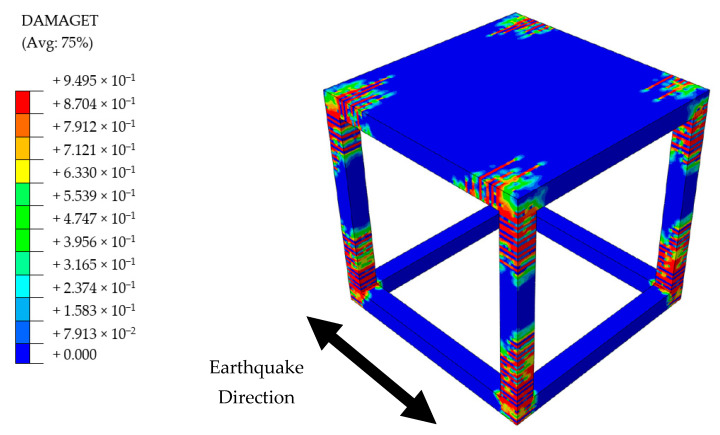
Damage distribution on the BF Building.

The BF building was able to sustain its overall strength until the end of the seismic loading. Typical plastic hinges were observed at the column ends. Such a failure is very common for buildings with limited lateral load carrying capacities. Since the BF type was modeled without the infill walls, a substantial portion of the earthquake caused forces needed to be carried out merely by the columns, which eventually led to damages to these parts. Besides, tensile cracks occurred in some parts of the top slab, where the beams located parallel to the earthquake direction connected to the columns. Distribution of the cracks at the last stage of analysis is presented in Figure 19.

**Figure 20:**
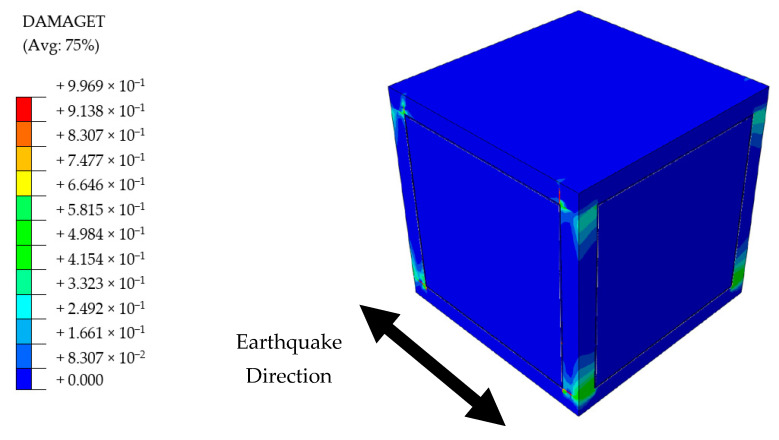
Damage distribution on the TM Building.

**Figure 21:**
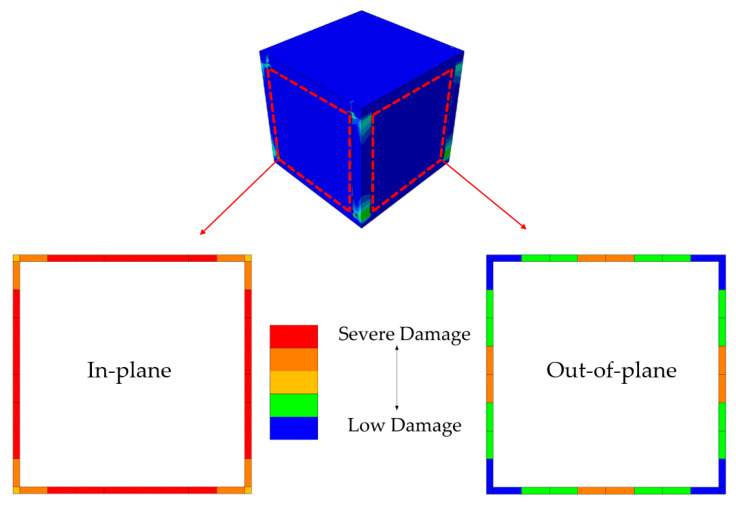
Mortar bonding failures for the TM Building.

TM building suffered severe damage in the vicinity of mortar joints parallel to the excitation direction, when the seismic loading reached to the peak acceleration of 16.0 m/s^2^ at the seconds of t: 4.03. Total loss of contact strength was experienced on these parts, hence the infill walls were not able to sustain a complete integrity with the rest of structural system. Therefore, the analysis was stopped at this point and the damages were investigated. Despite no major failure in the infill walls and low-to-moderate damage around the ends of RC column members, see Figure 20, the structural stability was nevertheless damaged due to the frame-to-wall bonding failure. In a real-life example, this situation would progressively lead to out-of-plane failure of the walls, even if a marginal overturning force is the concern. However, the walls that were placed perpendicular to the loading direction could withstand safely, although the middle region of mortar joints started to experience bonding strength decay. A schematic visualization of the contact damages is provided in Figure 21.

**Figure 22:**
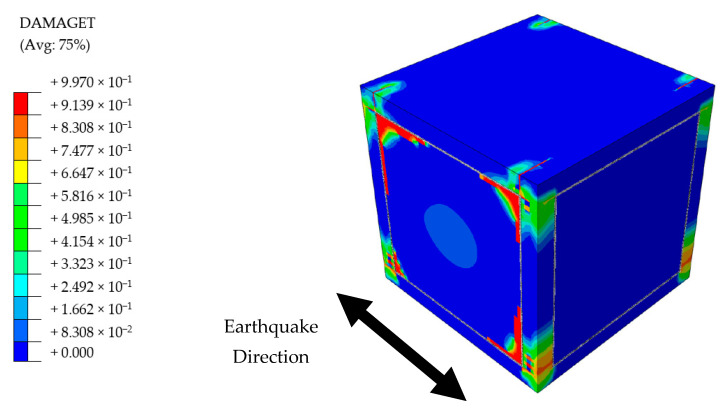
Damage distribution on the PUFJ Building.

Presence of the flexible joints made a visible contribution to the overall structural performance of PUFJ type of building. The system was able to survive the seismic loading without any substantial structural deficiencies, though some cracking damages were observed at the end of the earthquake. Among those, in-plane tensile forces induced cracks on the infill walls were the most visible. It is known from a previous experiment [36] that high bonding strength of the PolyUrethane PM might cause additional tensile forces on the walls due to the fact that the material resists the separation of different members i.e., frame and masonry. However, it is proven that such damages do not potentially harm the overall wall stability, since the largest portions of the joint surfaces remain bonded [36]. Regarding the RC frame, a similar pattern of damages of the other building types were observed, namely, tensile cracks around the column ends and on a limited portion of the top slab, see Figure 22.

**Figure 23:**
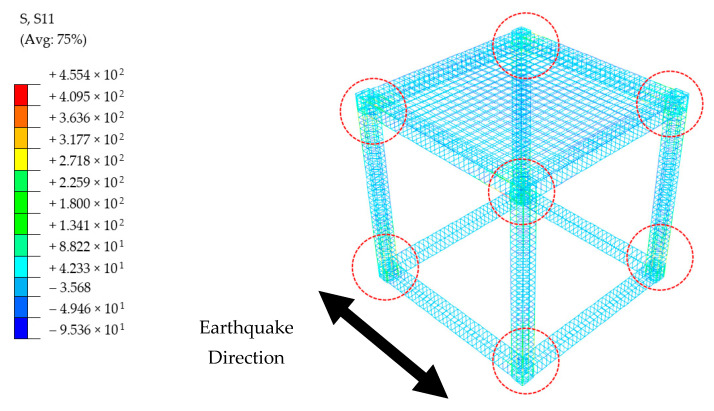
Reinforcement stresses belong to BF Building [MPa]; circles indicate the highest stress concentrations.

Practically no hazardous situations were spotted for any type of buildings when it comes to the subject of reinforcement bars only. The steel stresses were dominant in the tension region, and at the peak levels, were lower than the yield stress limit, 550 MPa. While it is worth mentioning that the BF building experienced the highest stress values around 450 MPa, both TM and PUFJ types had approximately 250 MPa stress at the maximum. In a similar way to the concrete cracking, aforementioned stresses were also located around the column ends and beam-to-column joints parallel to the loading direction. A representative view belongs to the BF building, and is given in Figure 23.

#### 3.4.2. Dynamic Characteristics

Dynamic behaviors of the buildings were evaluated in two ways by means of reviewing; modal frequency shifts as well as acceleration and displacement responses. Data for both different categories were taken for the whole structural systems, while omitting the individual material and element behaviors.

Eigen-frequency is a strong source for understanding the overall dynamic behaviors of structures. It enables us to identify the natural frequencies and corresponding mode shapes for the undisturbed oscillations. In the basic form and neglecting the damping forces, the equation below is well-known and can be written, where *fn*: natural frequency; *k:* stiffness and *m*: mass.

(2) $$\;f_{n}=\;\frac{1}{2\pi }\sqrt{\frac{k}{m}}$$

Equation (2) shows that the frequency is proportional to the square root of stiffness. The majority of civil engineering systems have relatively low damping features [59], which is around 5%. Therefore for such systems, *fd:* damped frequency converges to the natural frequency, *fn*, with a very good agreement, as shown in Equation (3), where *ζ* corresponds to the damping coefficient.

(3) $$f_{d}=f_{n}\;\sqrt{1-\zeta^{2}}$$

Considering Equations (2) and (3), a correlation is made between the flexural stiffness of structures and eigen frequencies, as given in Equation (4). *E* and *I* indicate the Young’s modulus and moment of inertia, respectively.

(4) $$\frac{EI_{current}}{EI_{initial}}=\frac{{\left(f_{current}\right)}^{2}}{{\left(f_{initial}\right)}^{2}}$$

**Figure 24:**
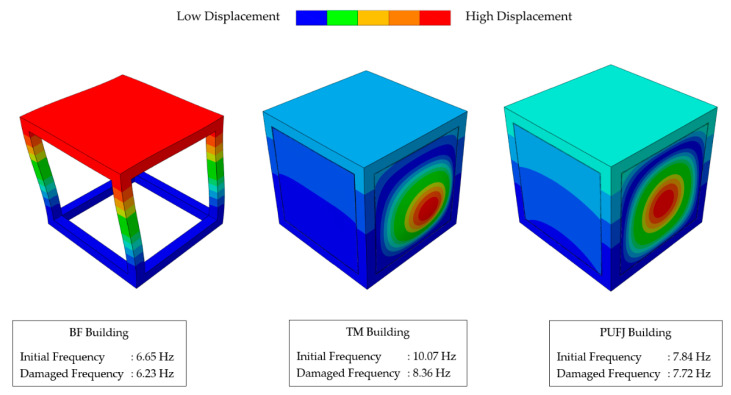
Mode shapes and frequencies of the buildings.

Frequency and stiffness change results of the buildings are given in Table 2. As previously mentioned and shown in Figure 18, the results were accumulated in two different stages, namely after the vertical loading (undamaged state) and at the end of earthquake loading (damaged state). BF building exhibited relatively ductile behavior compared to the other types, TM and PUFJ. This is an expected outcome, since the absence of infill walls caused a less stiff system, particularly in horizontal directions. Undamaged frequency of the BF building was measured as 6.65 Hz, whereas some reduction was observed at the end of analysis which led to frequency value at the damaged state as 6.23 Hz, corresponding to 93.7% of the initial state. Similarly, TM and PUFJ types of the buildings also experienced some drop in their natural frequencies at the end of analyses. TM building, thanks to the stiff connection type, had the highest frequency values of 10.07 Hz and 8.36 Hz for the undamaged and damaged states, respectively. As already mentioned in this paper, the analysis was stopped at some point due to the total connection loss of bonding regions between the masonries and RC frames. At the final moment of analysis, ratio between the damaged and undamaged frequency results were calculated as 83.1%. It shows a sharper decrease of frequency in comparison to the BF type. This result shows an interesting fact that, despite more intensive damages were observed in the BF building compared to the TM one, as can be seen in Figure 19 and Figure 20, such bonding failures might cause substantial instabilities on building dynamic characteristics. In this case, the wall detachment led to partial BF like behavior of TM building in the excitation direction, since the walls were not the parts of the structural system anymore. However, the effect of this failure was more severe in terms of the dynamic behavior, as the mass of walls still contributed to the inertia forces. However, frequency results of the PUFJ building were found between the ones of BF and TM types, as 7.84 Hz for the undamaged and 7.72 Hz for the damaged levels. The decrease of frequency value was marginal and calculated as 98.4% of the initial state. In accordance with the frequency results and assuming the undamaged conditions of buildings equal to 100% of their stiffness capacities, the stiffness changes were determined using the Equation (4). In this way, the effect of flexible joint implementation was rather visible, since the stiffness reduction was just slightly more than 3% and calculated as 96.9% of the undamaged state. Whereas the same trend of declination was very distinguishable for BF and TM types, 87.7% and 69.0% of the initial states, respectively. It is worth to mention that the system mass of BF type was approximately 20% less than the other two due to the absence of masonries, therefore the building was exposed to less inertial forces. Having the same amount of mass would potentially result higher level of damages. Furthermore, modal shapes of the buildings were examined and noticed that there was no considerable difference between the undamaged and damaged states for the dominant modes with the highest mass participation ratios. The mode shapes are given for the initial conditions in Figure 24.

**Figure 25:**
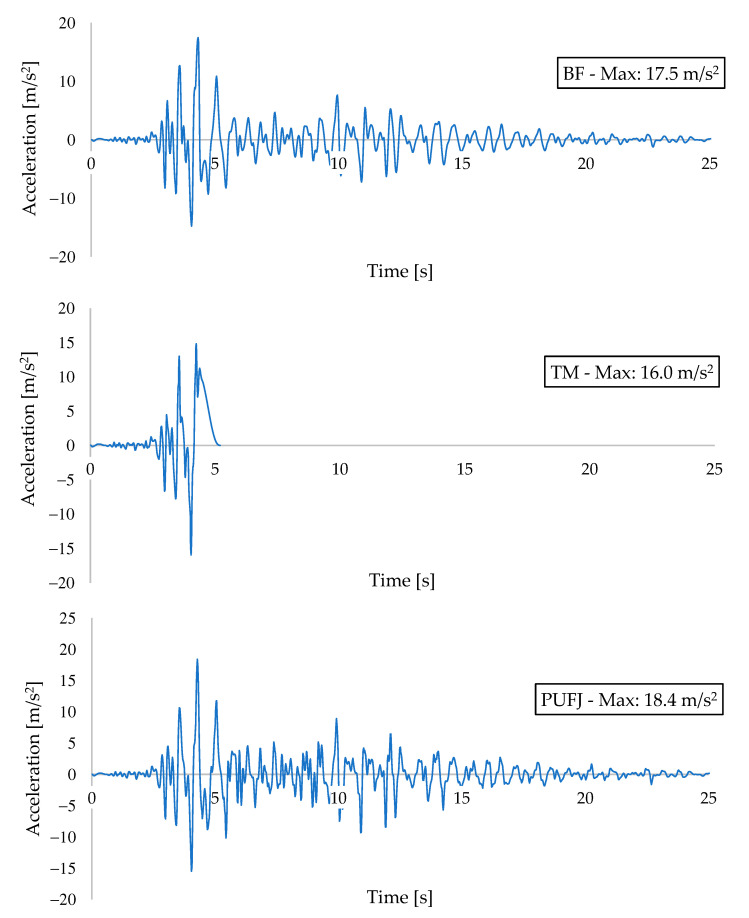
Acceleration-time outcomes of the buildings.

**Figure 26:**
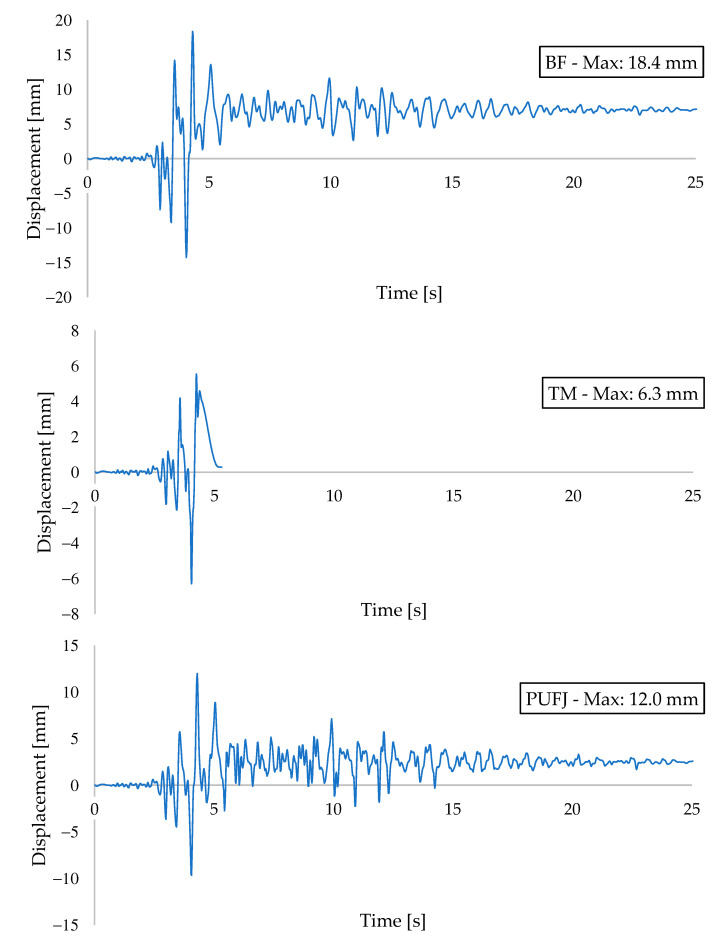
Displacement-time outcomes of the buildings.

Acceleration and displacement results are presented in Figure 25 and Figure 26, respectively. Top slab vertical coordinates were considered while extracting the acceleration data, whereas the relative difference between the middle points of the top and bottom beams were used to calculate the displacements throughout the earthquake loading. All of the buildings reached the highest acceleration and displacement values when the earthquake intensity approached peak levels, after the time of 4 s from the beginning. BF building experienced the maximum acceleration of 17.5 m/s^2^ at t: 4.32 s. Similarly, TM and PUFJ had the acceleration values as 16.0 m/s^2^ and 18.4 m/s^2^ at the times of t: 4.03 and t: 4.28 s, respectively.

When comparing the displacement outcomes, it is seen rather distinct difference between the results. BF had the maximum displacement as 18.4 mm, which was greater than the same findings of TM and PUFJ types; 6.3 mm and 12.0 mm, respectively. Corresponding maximum drift ratios were calculated for BF: 0.56%, TM: 0.19% and PUFJ: 0.36%. The results can be interpreted as a BF building, which exhibited the most ductile behavior due to the absence of infill walls. However, the TM type had limited capacity of lateral displacement and could therefore withstand only the maximum value of one-third of BF and half of the PUFJ approximately. Moreover, the highest acceleration and therefore inertia forces were absorbed by the PUFJ building. It is a sign that flexible joints contribute to the lateral load carrying capacity considerably, with a reasonable drift ratio. Table 3 presents the acceleration and displacement outcomes.

## 4. Discussion and Conclusions

Researchers from all around the world work on several solutions in order to mitigate the earthquake hazards on buildings [60,61,62,63]. Polymer based flexible joint approach, PUFJ, was investigated numerically by means of utilizing various methods. Guidelines related to modeling of earthquake hazards are clearly described in Reference [64]. Dynamic analyses are often considered to be the most reliable tools, while aiming to reveal the building performances under seismic loads. Accordingly, three-dimensional real-size specimens were created in the finite elements method environment and exposed to the earthquake loads. The main outcomes are given below.

- Large size three-dimensional problems require much information to be taken into account. Therefore, small size specimens were first numerically created in order to obtain an accurate material data for further analyses. Previous experimental results [52] were used while calibrating the models. Once the models provided adequate convergence with the test results, simplified macro wallet tests were performed numerically using the same data. It is shown that PUFJ can be modeled numerically and this approach yields a close match with the test results.
- After this point, the study proceeded by large size models. The structures were biaxially symmetric, hence the seismic excitation was performed only in one direction. Damage levels of each material were investigated at the end of earthquake loading. BF and PUFJ buildings were able to withstand earthquake effects until the end of loading. Meanwhile, TM building suffered severe bonding failure of the mortar around the entire perimeter of masonries, which were located parallel to the loading direction. Especially in real life examples, such in-plane damages progressively cause the total loss of connection strength of the walls and therefore out-of-plane failure is inevitable. Other than that, and excluding the bonding failure in TM building, masonries sustained the internal integrity in both TM and PUFJ buildings, although some tensile corner cracks were observed in the PUFJ type due to the strong bonding features of the polymer. However, it is seen that such damages do not jeopardize the overall structural performance, which was also proven elsewhere [36]. Moreover, all building types experienced concrete cracks in particular regions, mostly concentrated at the column ends as expected. Reinforcement steel did not pass beyond the elastic range, despite the highest stresses were more intense in the vicinity of aforementioned column ends.
- Frequency analyses were conducted at two stages; at the beginning of horizontal loading for understanding the undamaged state conditions and at the end of earthquake loading for representing the damaged state. Accordingly, TM building had the highest initial frequency value due to the stiff connection around the infill walls, whereas the BF type had the lowest one since there were no walls in this type, which could contribute to the lateral load carrying capacity. The effect of flexible joint implementation was explicit at the damaged states. PUFJ building had very little drop of frequency, meanwhile particularly TM type had relatively harsher frequency reduction. This comparison was more visible when the frequency-based stiffness changes were evaluated. TM building had significant loss of stiffness capacity, reaching to 30% of the initial value. The frequencies obtained during the study have been checked with the results of other researches [65,66]. In Reference [65], the investigated building was damaged, and the measurements and model were made. First natural frequency was equal about 5.5 Hz, but the building was a little bit higher than the structure investigated in the paper. In [66], one-storey undamaged monumental building was investigated. The first natural frequency was equal about 6.5 Hz, which is quite similar to the results listed in Table 2. Furthermore, even though it was not examined in this paper, efficiency of the PUFJ material was tested in [67] against the resonance frequencies. Geometric configuration of the building was very similar of the PUFJ type of building presented in this study, namely a real-size single storey RC structure comprised of brick walls bonded to the frame with PUFJ [37]. It was revealed that various intensities of long-duration (up to 10 min) forced harmonic vibrations were unable to collapse the tested structure, which was previously exposed to the shake table vibrations and was therefore already damaged.
- Acceleration and displacement data was accumulated throughout the loading. The BF building had the highest top slab displacement values and it was visibly the most ductile one among the others. PUFJ type, however, was exposed to the greatest acceleration forces and was able to damp this energy safely. TM building had the lowest peak values of both acceleration and displacement when compared to the other buildings. The system response was weakened due to the bonding failures of the mortar located between the masonries and RC frame.
- As a result of this numerical study and according to the outcomes of previous experimental tests [36,37] of PUFJ, it is seen that the infill wall stability can be sustained even under severe loads. Unlike some proposals, which can be found in the literature and already mentioned in the introduction part of this paper, PUFJ claims to offer a solution to protect the in filled systems against the earthquakes, while at the same time contributing to the drift and strength capacity of the overall system. Implementation of PUFJ is also rather feasible compared to the typical CFRP strips, since any contact surface between the frames and walls can provide sufficient and effective bonding. It can be used on both existing buildings and new to-be-built constructions. For the already built walls, implementation can be done by means of cutting the edges of masonries and injecting the liquid form of polymer in the remained gaps, whereas prefabricated laminates are ideal to be used for the new buildings, which should be placed on the boundaries of frames just before constructing the walls. Implementation details can be found in [36,37]. On the other hand, as previously mentioned, some damages on the wall itself rather than the contact zones might be observed in case of PUFJ implementation. This situation was seen in the experimental tests [36], where hollow-clay bricks were used and also in the numerical analyses of this paper, which predicted potential tensile cracks on the solid clay masonry. While the damages were relatively less compared to the stiff jointed frames and do not seem to pose any risk to the wall stability, further studies on this topic should focus on testing other masonry materials, brick types and different configurations, such as aspect ratios and openings on the walls.

All in all, infill walls are largely preferred across the globe and these are therefore expected to either contribute building load carrying capacity or at least not present any potential risks as being passive structural members. This study revealed that the preferred bonding method between walls and frames indeed has a substantial effect on the structural behavior. Earthquakes release enormous energy and stiff connections might very often fail to provide sufficient capacity. In such cases, masonries affect the buildings detrimentally, despite these members being constructed with completely good intentions. The PUFJ approach, in this sense, provides a promising solution to be used in seismic zones.

## Figures and Tables

**Table 1:** Material properties of brick units, concrete, mortar and masonry.

	Elastic Properties		CDP Properties				
	*E* [Mpa]	*ν*	Ψ	*ε*	*σ_b_*0/*σ_c_*0	Kc	Viscosity Parameter
Brick Unit	4500	0.15	30	0.1	1.16	0.67	0.001
Concrete	35,000	0.2	30	0.1	1.16	0.67	0.0001
Mortar	1400	0.2	30	0.1	1.16	0.67	0.0001
Masonry	1000	0.25	30	0.1	1.16	0.67	0.05
*E*	Young’s Modulus						
*ν*	Poisson’s Ratio						
Ψ	Dilatation Angle						
*ε*	Flow potential eccentricity						
*σ_b_*0/*σ_c_*0	Equibiaxial to uniaxial compressive yield stress ratio						
Kc	Stress invariant ratio						

**Table 2:** Frequency and stiffness changes of the buildings.

	Frequencies [Hz]			Stiffness Change [Undamaged Equals to 1.00]		
	BF	TM	PUFJ	BF	TM	PUFJ
Undamaged	6.65	10.07	7.84	1.00	1.00	1.00
Damaged	6.23	8.36	7.72	0.88	0.69	0.97
Ratio	93.7%	83.1%	98.4%	87.7%	69.0%	96.9%

**Table 3:** Acceleration and displacement responses of the buildings.

	BF	TM	PUFJ
Maximum Acceleration [m/s^2^]	17.5	16.0	18.4
Maximum Displacement [mm]	18.4	6.3	12.0
Maximum Drift Ratio [%]	0.56%	0.19%	0.36%

## Data Availability

The data presented in this study are available on request from the corresponding author.
